# Tracheal intubation with video laryngoscopy in out-of-hospital sitting

**DOI:** 10.1186/s13049-021-00913-2

**Published:** 2021-10-04

**Authors:** Bin Hu, Tian Tian, Fu-Shan Xue

**Affiliations:** Department of Anesthesiology, Beijing Friendship Hospital, Capital Medical University, NO. 95 Yong-An Road, Xi-Cheng District, Beijing, 100050 People’s Republic of China

**To the Editor**

With great interest we read the article written by Knapp et al. [1] recently published in Scandinavian Journal of Trauma, Resuscitation and Emergency Medicine. They showed that use of C-MAC video laryngoscope by operators with mixed experience provided a high first-pass intubation success (FPS). Given that safe airway management is very important for successful resuscitation of critically ill or injured patients in out-of-hospital sitting and the benefits of video laryngoscopes are often significant in airway management of out-of-hospital emergency patients [2], their findings have potential implications. However, there were two methodological issues in their article on which we invited the authors to comment.

First, primary outcome of this study was the FPS. However, an important question neglected by authors is that C-MAC video laryngoscope is a Macintosh-type device, with ability to perform both direct and video laryngoscopy using same device. That is, the larynx can be seen either under direct vision or on a monitor when using C-MAC device [3]. This advantage of C-MAC device makes it exceptionally useful for emergency intubation. For example, in the event of a failed video laryngoscopy attempt due to fogging of the lens, bloods and secretions on the camera and direct solar irradiation on the screen, intubators can immediately switch direct laryngoscopy to continuously perform intubation procedure without the need to remove device from the mouth for a second attempt, and vice versa [4]. In fact, after a failed first attempt with an impeded view in this study, intubators also changed from video laryngoscopy to direct laryngoscopy (still using C-MAC device) for successful intubation. Thus, it was unclear why this situation was defined as a second attempt. We completely agree with Sakles et al. that FPS is a useful endpoint for assessing intubation performance of direct laryngoscopy or angulated video laryngoscopy, but not for Macintosh-type video laryngoscopy [4].

Second, this study showed no correlations between intubators’ work experience and performance variables of C-MAC device, such as FPS, overall success rate, intubation time and intubation difficulty level. This is not in accord with the recent findings of Amalric et al’ study [5], in which the operators’ expertise, which is assessed by number of previous video laryngoscopies performed, is an independent determinant of FPS with McGrath MAC video laryngoscope in critically ill patients. Most important, it was unclear what statistical method was used to determine correlations between intubators’ experience and performance variables of C-MAC device in this study. To determine independent effect of intubators’ experience on performance of C-MAC video laryngoscope, we argue that multivariate analysis should be used for adjusting patients’ baseline characteristic and controlling selection biases.

## Response letter on the letter to the editor

Jürgen Knapp^1*^, Bettina Eberle^1,2^, Michael Bernhard^3^, Lorenz Theiler^4,5,^ Urs Pietsch^5,6^ and Roland Albrecht^5,6^

juergen.knapp3@googlemail.com

^1^Department of Anaesthesiology and Pain Medicine, Inselspital, Bern University Hospital, University of Bern, Bern, Switzerland

^2^Department of Anaesthesiology, Cantonal Hospital of Graubünden, Chur, Switzerland

^3^Emergency Department, Heinrich-Heine-University, University Hospital of Düsseldorf, Düsseldorf, Germany

^4^Department of Anaesthesiology, Cantonal Hospital of Aargau, Aarau, Switzerland.

^5^Swiss Air Rescue, Rega, Zurich, Switzerland

^6^Department of Anaesthesiology and Intensive Care Medicine, Cantonal Hospital St. Gallen, St. Gallen, Switzerland

We thank Hu et al. for their letter commenting on our study and for their friendly acknowledgment of our results [1, 6]. Here, we gladly answer their questions:

- After a failed attempt with impeded view, intubators were allowed to change from video to direct laryngoscopy, still using the C-MAC device. However, this was not defined as a second attempt as long as the intubator did not remove the blade from the patient’s mouth. We apologize for not stating this clearly in our paper.

Second, Hu et al. suggest performance of a multivariate analysis to adjust for patients’ baseline characteristics. We did not perform this analysis in our current analysis and therefore our results do not allow the conclusion that operator experience is not an independent determinant of first-pass success. However, our results clearly show that in this “real life” observational study, inexperienced operators perform as well as very experienced operators. Therefore, our conclusion only states that the use of a C-MAC video laryngoscope “seems to be beneficial in a group of providers with very variable expertise in airway management” [1]. Currently, we are performing an analysis of the learning curve for video laryngoscopic-assisted tracheal intubation in a much larger setting.

## Data Availability

Data sharing not applicable to this article as no datasets were generated or analyzed during the current study.
